# Supplementary material for: Dual Functionalized Liposomes for Selective Delivery of Poorly Soluble Drugs to Inflamed Brain Regions
Source: Pharmaceutics. 2022 Nov 7;14(11):2402. doi: 10.3390/pharmaceutics14112402 (PMC9698607; doi:10.3390/pharmaceutics14112402)
Supplement: Supplementary file 1 [file pharmaceutics-14-02402-s001.zip › pharmaceutics-1959574-supplementary.pdf]

Supplemental Materials and Methods

Synthesis of Fmoc-Lys(stearate)-OH

To an ice-cold solution of Fmoc-Lys(Boc)-OH (10.68 mmol, 5 g, 1 eq) in DCM (30 ml), TFA (106.8 mmol, 8 ml, 10 eq) was added dropwise. After 10 min, the ice bath was removed and the resulting solution was stirred at RT for 2 h. Then, the volatiles were evaporated *in vacuo* and the oily residue was triturated with Et_2O. The white precipitate was filtered and dried to afford Fmoc-Lys-OH (3.8 g, 94% yield). Without any further purification to an ice-cold solution of Fmoc-Lys-OH (2.08 mmol, 1.0 g) in DCM/DMF 5:1 (7 ml), DIPEA (2.10 mmol, 0.37 ml) was added dropwise. Meanwhile, in a second flask containing a solution of stearic acid (1.1 eq) in DCM/DMF 5:1 (4 ml), EtCN-oxime (1.1 eq) and EDC (1.1 eq) were added respectively and the reaction mixture was stirred at RT for 30 min. The activated acid was added to the Fmoc-Lys-OH at 0° C, stirred for 10 min at this temperature and then at RT until completion of reaction (3-5 hours). The reaction mixture was diluted with DCM and washed with 5% aqueous $KHSO_4$ solution and brine. The organic phase was dried over anhydrous Na_2SO_4 and evaporated *in vacuo*. The residue was triturated with Et_2O. The pale-yellow precipitate was filtered and dried to afford Fmoc-Lys(stearate)-OH in 66% yield. MS-ESI m/z 635.20 $[M+H]^+$, 657.42 $[M+Na]^+$, 1291.15 $[2M+Na]^+$. ^{1}H NMR (300 MHz, $CDCl_3$) δ 7.76 (d, *J* = 7.2 Hz, 2H), 7.59 (s, 2H), 7.45 – 7.21 (m, 4H), 5.79 – 5.55 (m, 2H), 4.38 (s, 2H), 4.22 (d, *J* = 6.2 Hz, 2H), 3.38 – 2.61 (m, 6H), 2.23 – 2.03 (m, 3H), 1.69 – 1.10 (m, 34H). The reported data are according to literature[1].

HPLC chromatography

HPLC chromatograms were recorded on JASCO HPLC, furnished of AS4150 autosampler, PU-4180 pumps, CO4061 oven and MD-4010 PDA detector. Chromatogram are reported in function of absorbance at 220nm (A_{220}) and time (min). MSPL were analysed on a Phenomenex column, Kinetex 2, C18, 6µm, 100A, by using RP conditions: phase A (1% TFA in water), phase B (1% TFA in ACN). Method: 20-100% ACN in 18 min: 0-3 min 20% B, 3-11 min 80% B, 11-21 min 100% B, 21-26 min 100%B.

Evaluation of MSLPs structure by circular dichroism

The circular dichroism (CD) spectra were recorded on a Jasco J-810 spectropolarimeter, using a quartz cell with a path length of 0.1 cm. Spectra were recorded with the following parameters: 10 nm/min scan speed, 1 nm band width, 1 s collection time per step, scanning mode continuous, 100 mdeg sensitivity and 3 repeats. The CD signal from the background (TFE, TFE/water 1:1, TFE/PBS buffer) was subtracted from the CD signal of the sample solution. Ellipticity is reported as the mean residue ellipticity ([θ], in deg cm^2/dmol) and calculated as $[\theta] = [\theta]_{obs}\frac{MRW}{10cl}$, where $[\theta]_{obs}$ is the ellipticity measured in millidegrees, MRW is the mean residue molecular weight of the peptide (molecular weight divided by the number of amino acid residues), *c* is the concentration of the sample in mg/mL, and *l* is the optical path length of the cell in centimetres. Samples

were measured using quartz cells with 0.1 cm length. MSLPs were dissolved in TFE and TFE/water 1:1 at a final concentration of 0.2 mg/ml (final volume contained in the quartz cell: 400 µl). For characterization of peptides in the presence of liposome-simulating conditions, sphingomyelin and cholesterol were dissolved in 1:1 ratio in TFE/PBS buffer 1:1 mixture. Spectra were acquired with a peptide concentration of 0.1 mg/ml, and a liposome concentration of 1 mg/ml, using TFE/PBS buffer 1:1 as solvent. Secondary structure analysis has been performed using Dichroweb programme[2]. Data were obtained using the following parameters: 1) for Contin-LL (Provencher & Glockner Method: Reference set SMP180, 190-240 nm): millidegrees/theta as input units, 250 nm as initial wavelength, 190 nm as final wavelength, 0.1 nm as wavelength step, 190 nm as lowest nm data point and mean residue ellipticity as output units; 2) for K2d Method (wavelength range 200-240 nm): millidegrees/theta as input units, 250 nm as initial wavelength, 190 nm as final wavelength, 0.1 nm as wavelength step, 200 nm as lowest nm data point and mean residue ellipticity as output units.

References

1. Zhang, L., Robertson, C. R., Green, B. R., Pruess, T. H., White, H. S., and Bulaj, G. (2009) Structural Requirements for a Lipoamino Acid in Modulating the Anticonvulsant Activities of Systemically Active Galanin Analogues. *J. Med. Chem. 52*, 1310–1316.

2. Abdul-Gader, A., Miles, A. J., and Wallace, B. A. (2011) A reference dataset for the analyses of membrane protein secondary structures and transmembrane residues using circular dichroism spectroscopy. *Bioinformatics 27*, 1630–1636.

Supplementary Figures

FIGURE S1

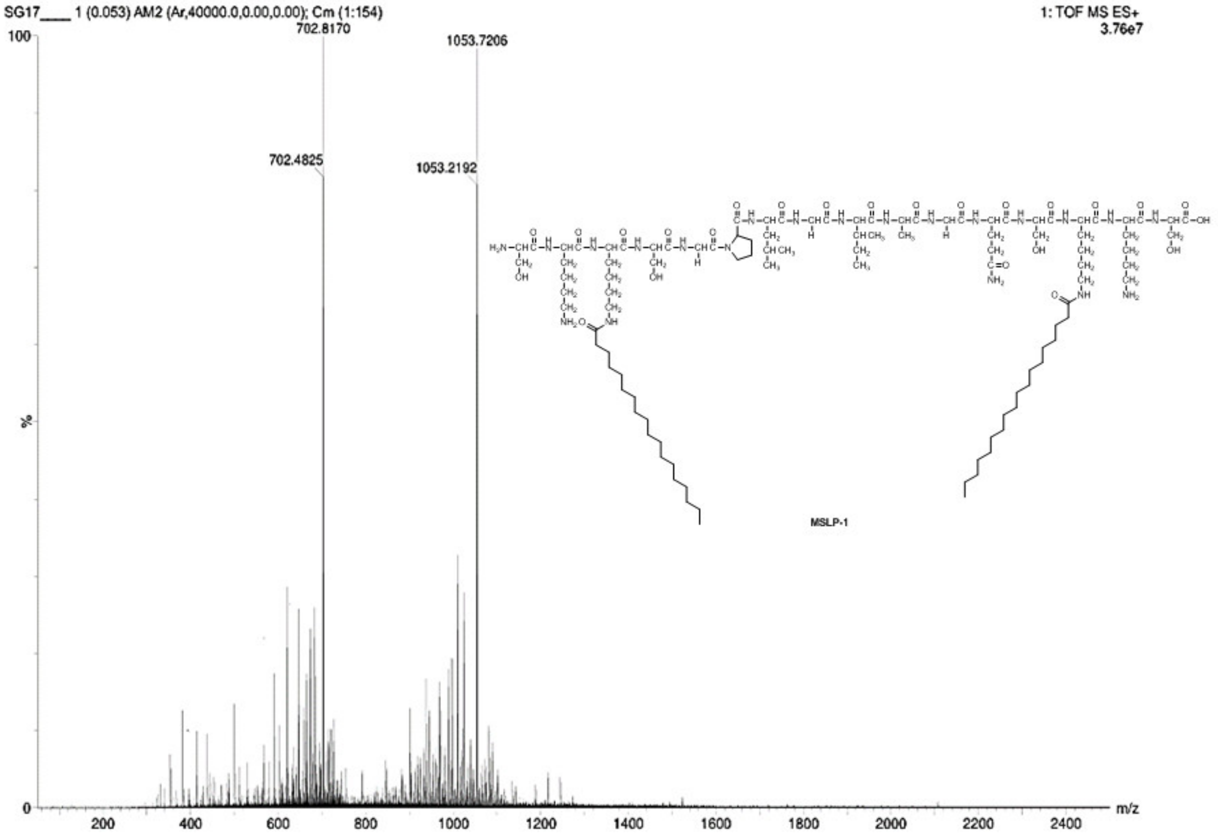

Figure S1. Chemical structure of **MSLP-1.** HRMS-ESI m/z: 1503.7206 $[M+2H]^{2+}$, 702.8170 $[M+3H]^{3+}$

FIGURE S2

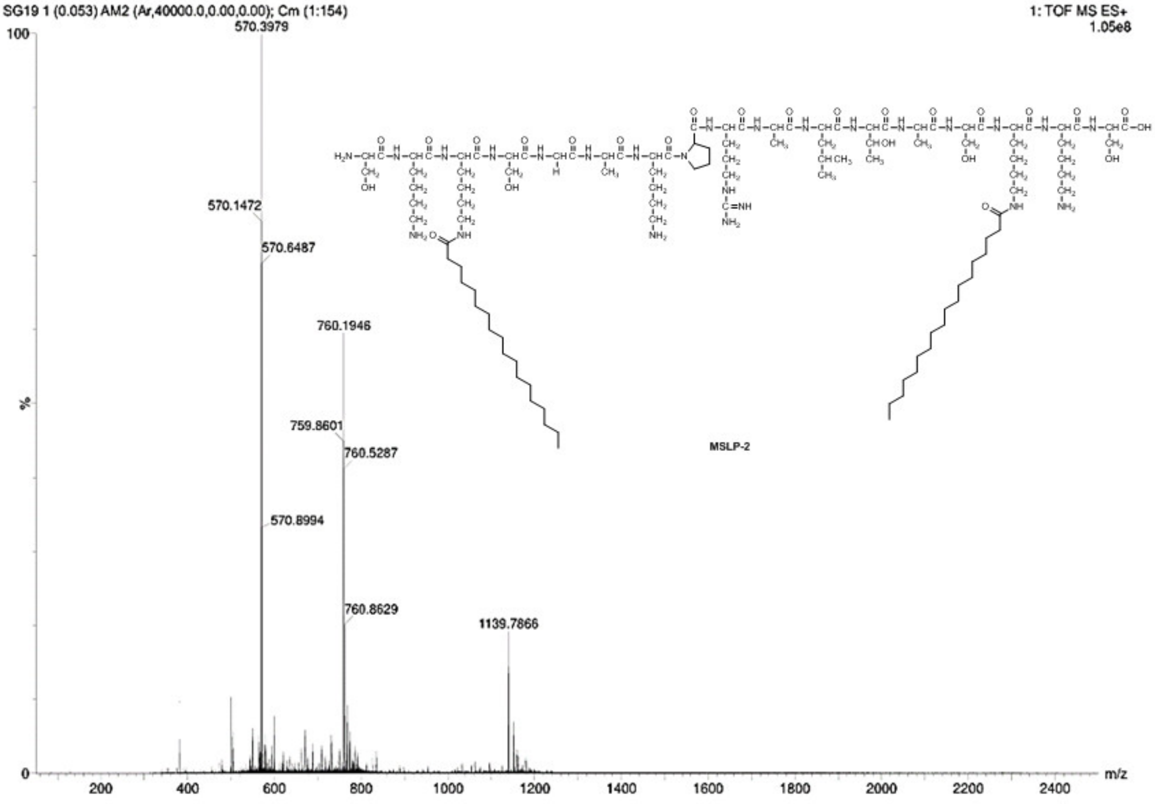

Figure S2. Chemical structure of **MSLP-2.** HRMS-ESI m/z: 1139.7866 $[M+2H]^{2+}$, 760.1946 $[M+3H]^{3+}$, 570.3979 $[M+4H]^{4+}$.

FIGURE S3

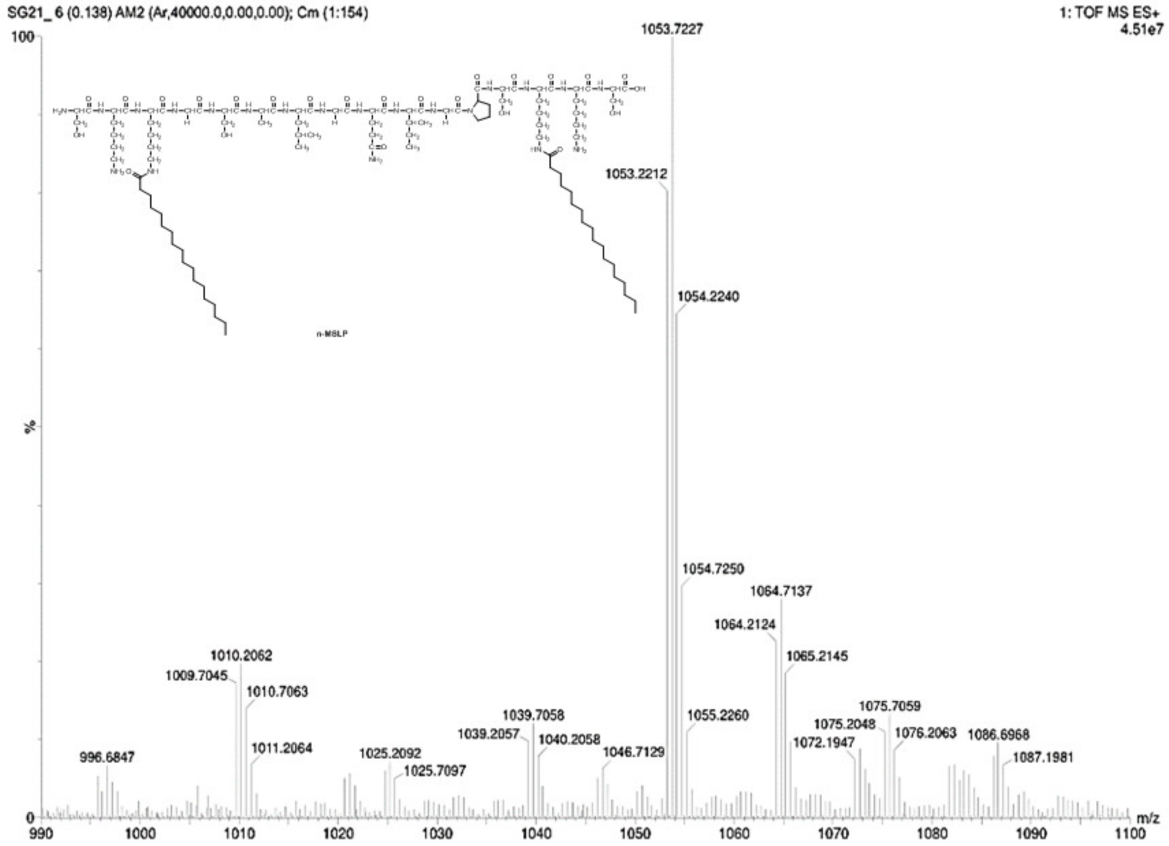

Figure S3. Chemical structure of **c-MSLP.** HRMS-ESI m/z: 1503.7227 $[M+2H]^{2+}$

FIGURE S4

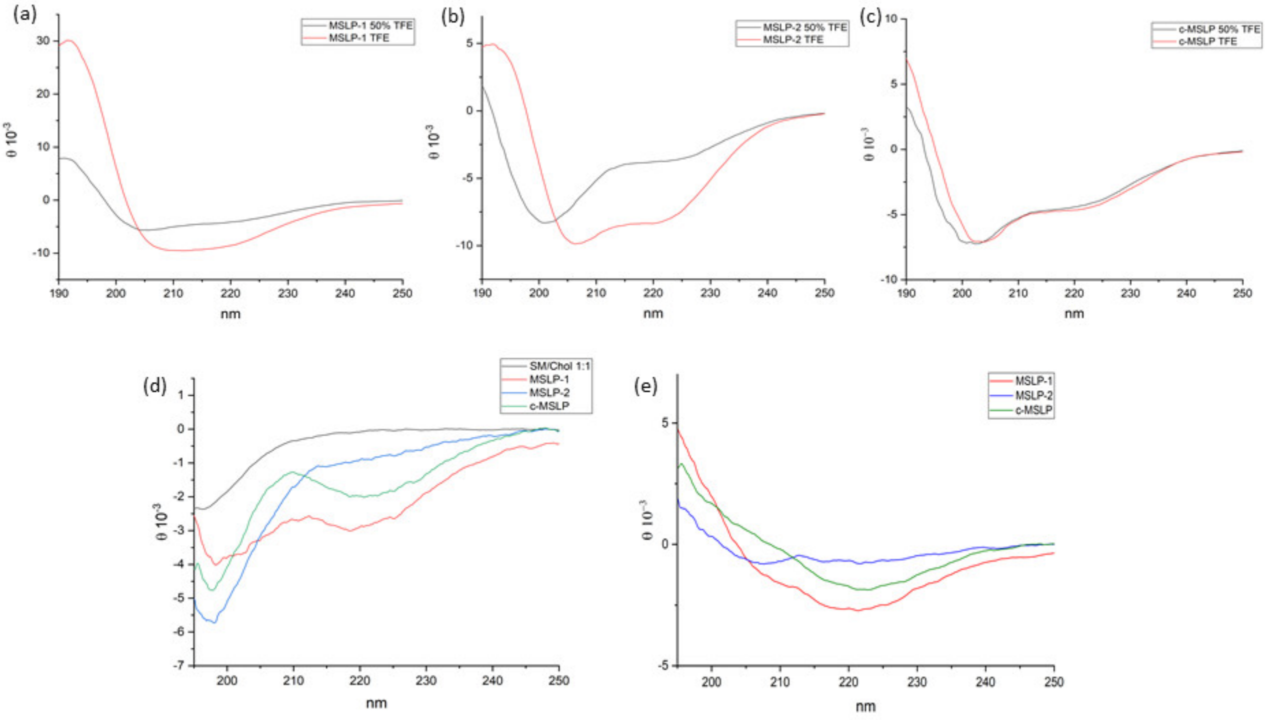

Figure S4. Circular dichroism analysis. Mean residue ellipticity of MSLPs at 200 µM in TFE and 50% TFE in water (free conditions). (a) MSLP-1; (b) MSLP-2; (c) c-MSLP. Mean residue ellipticity of the corresponding solution of MSLPs at 100 µM in TFE/PBS buffer 1:1, in the presence of Sm/Chol 1:1 at the concentration of 1 mg/mL. (e) CD spectra of MSLPs/Sm/Chol and SM/Chol; (d) CD spectra of MSLPs subtracted from CD spectra of SM/Chol.

FIGURE S5

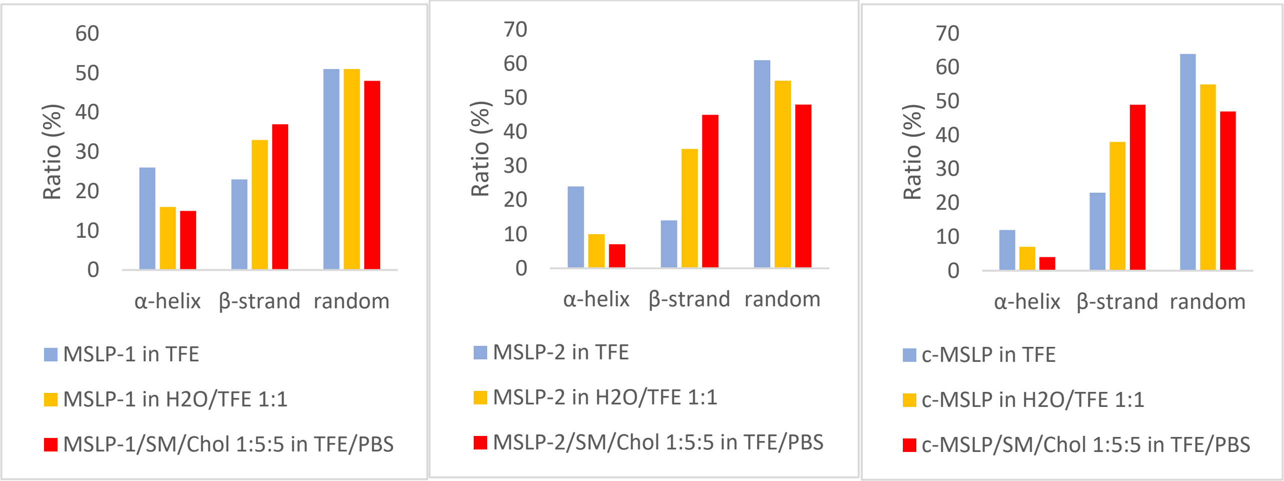

Figure S5. Conformation analysis. Secondary structure percentage data obtained using Dichroweb. Data regarding the CD spectra of MSLPs in the presence of SM/Chol have been calculated with K2d method instead of Contin-LL (Provencher & Glockner Method: Reference set SMP180, 190-240 nm), due to the narrower wavelength range used for the calculation, 200-240 nm.

FIGURE S6

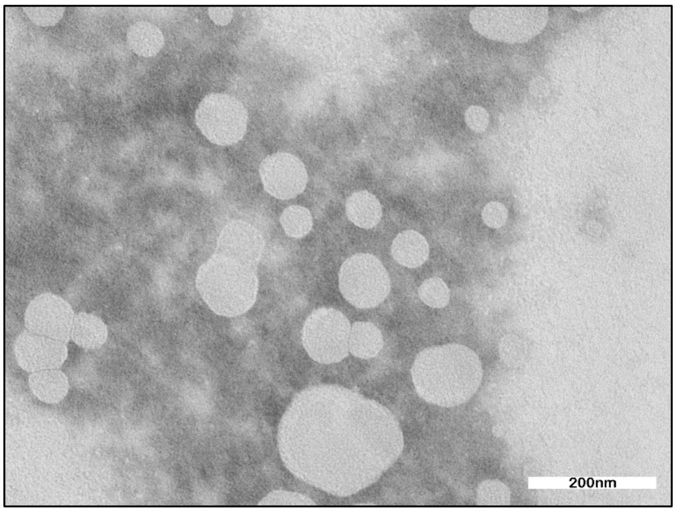

Figure S6. Representative TEM image of diluted MSLP-liposomes (scale bar 200 nm).

FIGURE S7

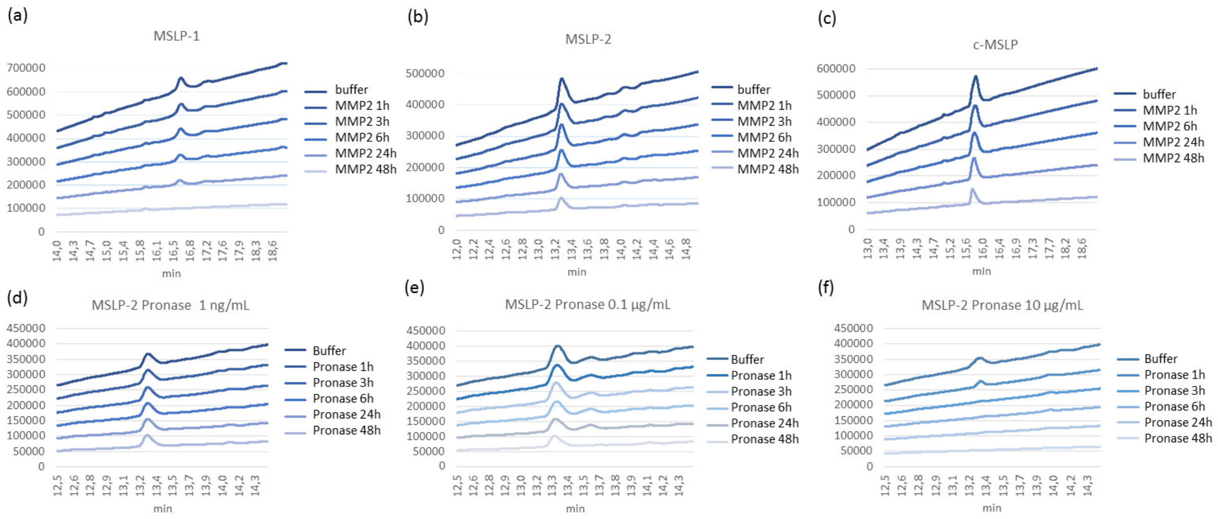

Figure S7. MMP2 activity assay on MSLPs. Cleavage of MSLP-1 (a), MSLP-2 (b), c-MSLP (c) was monitored through HPLC at different time points (1h, 3h, 6h, 12h, 24h, 48h). Pronase activity assay on MSLP-2, using enzyme different concentration: 1 ng/ml (d), 0.2 µg/ml (e) and 20 µg/mL (f). Cleavage of MSLP-2 was monitored through HPLC at different time points (1h, 3h, 6h, 12h, 24h, 48h).

FIGURE S8

(a)

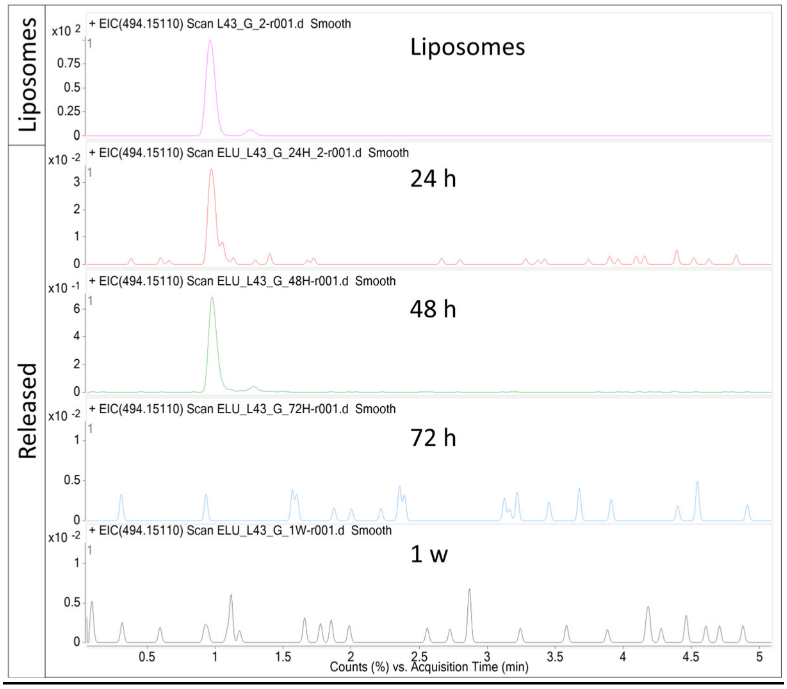

(b)

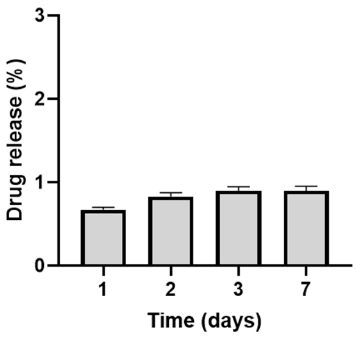

Figure S8. Release of Glibenclamide from MSLP-liposomes over time in the absence of MMPs, assessed by LC-MS. (a) Extracted Ion Chromatograms (EICs) of Glibenclamide; (b) drug released from liposomes over time, expressed as percentage of total embedded Glibenclamide. Data are expressed as mean ± SD.

FIGURE S9

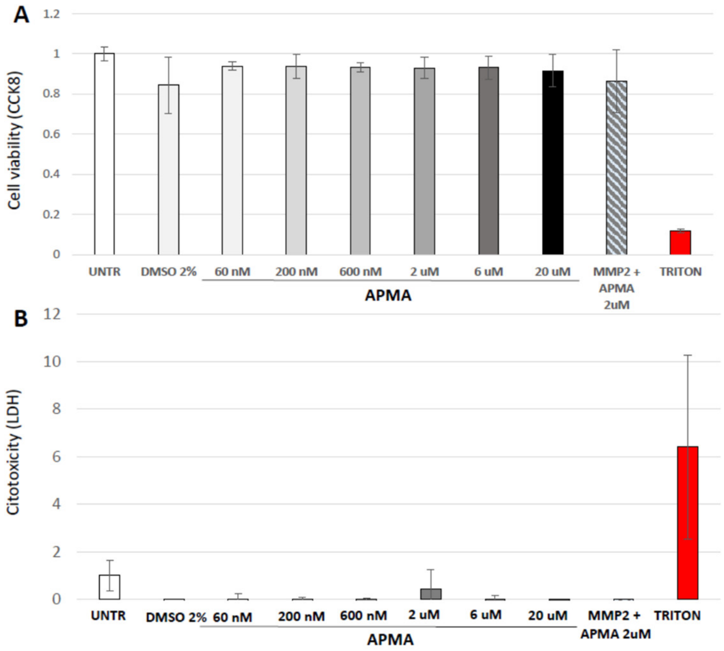

Figure S9. BV2 cells treated with: DMSO 2%, p-aminophenylmercuric acetate (APMA) at different concentrations (from 60nM to 20uM) and APMA 2uM in combination with MMP2 3nM; Cells treated with Triton X-100 were used as positive controls for LDH assay. (A) Cell viability evaluated with Cell Counting Kit - 8 (CCK8) metabolic assay, performed after 24h of treatments. Sample readouts were normalized to untreated. (B) Cell mortality evaluated with LDH-Glo Cytotoxicity Assay, performed after 24h of treatments. Sample readouts were normalized to untreated.
